# Rubber Hand Illusion Reduces Discomfort Caused by Cold Stimulus

**DOI:** 10.1371/journal.pone.0109909

**Published:** 2014-10-08

**Authors:** Marta Siedlecka, Anna Klimza, Marta Łukowska, Michał Wierzchoń

**Affiliations:** 1 Consciousness Lab, Institute of Psychology, Jagiellonian University, Krakow, Poland; 2 Faculty in Wroclaw, University of Social Sciences and Humanities, Wroclaw, Poland; University of London, United Kingdom

## Abstract

There is a growing interest in body-ownership disruptions and their consequences for subjective experiences such as tactile sensations or pain. Here, we investigated the effect of the rubber hand illusion (RHI) on the perceived discomfort caused by cold stimulus applied to the real hand. The results showed reduced discomfort to cold reflected in behavioural and subjective measures. The stronger the illusion, the later the cold temperature became unpleasant and the less intense the experience was rated. We discuss the link between thermoception and body ownership as well as possible theoretical and methodological implications for studies on pain experience under RHI.

## Introduction

The sense of body-ownership refers to the feeling that a person's body belongs to them [1]. The rubber hand illusion (RHI) [2] is an experimental way of altering this feeling by inducing a conflict between visual, tactile and proprioceptive information. In a typical RHI protocol a participant's experimental hand is hidden from view and stroked synchronously with a visible rubber hand. As a result the participant usually experiences tactile sensations as coming from the rubber hand and misjudges the position of the unseen real hand.

Subjective, behavioural, physiological and brain imaging data suggest that during the RHI the artificial hand (or hands) becomes a part of body representation [3]–[6]. However, little is known about how taking ownership of the rubber hand affects the real arm. It has been suggested that during the RHI the real hand becomes to some extent excluded from the body representation, both in terms of phenomenal experience and physiological regulation [7]–[12]. Although participants do not always report a strong feeling of real hand disownership [9], [18], the change of body representation might result in an absence of the real hand from participants' experience [14]. Moreover, Moseley and colleagues [10] (see also [8]) found that altering the sense of hand ownership during the RHI reduced skin temperature in this hand (but not in the other hand). Although this effect is not always detected [13], it is in accordance with clinical data showing a lower temperature in the affected limb in patients suffering body ownership disruptions such as self-mutilation disorders and complex regional pain syndrome [15]–[17]. Moseley and colleagues also showed that during RHI trials tactile stimulation from the experimental hand was processed slower than from the other hand (i.e. the experimental hand had to be stimulated first in order for two stimuli to be perceived as simultaneous). This finding was supported by Folegatti and colleagues [18] who observed longer reaction times to tactile stimuli delivered to the experimental hand under the RHI compared to a control condition. It was also shown that the RHI induces higher histamine reactivity in the “excluded” arm, a response observed in autoimmune disorders [19].

Here we aimed to further investigate the effect of the RHI on the real hand and find out whether inducing the sense of rubber hand ownership results in decreased discomfort to a cold, unpleasant stimulus applied to the real hand. To the best of our knowledge, only three studies investigated the effect of the RHI on temperature sensitivity in the real hand, with inconsistent results. In two rigorously controlled experiments Mohan and colleagues [20] attached a small heat probe to participants' experimental hand and did not find any differences in thermal pain intensity, thermal pain thresholds nor temperature perception thresholds in the real hand before and after the RHI induction. Valenzuela-Moguillansky and colleagues [12] conducted two experiments on thermal pain intensity and obtained conflicting results, which were attributed to several differences between experimental plans and set-ups. Most importantly, there was a discrepancy between the control conditions (non-stroking in Experiment 1 and asynchronous stroking in Experiment 2) that resulted in a different degree of rubber and real hand ownership between both experiments. Thermal pain ratings were slightly reduced in the experimental condition in which participants experienced stronger disownership of the real hand compared to the control condition (Exp. 1). When manipulation altered only the sense of rubber hand ownership, the pain intensity did not differ between conditions (Exp. 2).

Recently, a study conducted by Hegedüs and colleagues [21] showed an increased thermal pain threshold for hot stimuli in the real hand but no effect of the RHI on pain ratings. The authors claim that one of the main methodological differences between their study and the previous experiments is that participants placed their fingers on the heat probe built into the table. Therefore, contrary to the previous studies [12], [20], the strokes and the noxious stimuli were applied exactly to the same part of the hand. Secondly, the probe was not visually salient, and therefore the possibility of “visual capture of pain” and referral of pain to the rubber hand might have been reduced. It is important to note that in the previous studies on thermal pain during the RHI [12], [20] the heat probe was attached to both real and rubber hand. This raises the possibility that participants might have expected the painful stimuli to be applied to the rubber hand, perceived as their own body part. Studies show that threatening the rubber hand during the illusion elicits physiological and neuronal responses similar to those evoked by a threat to a real body part and that the magnitude of those responses correlates with the strength of rubber hand ownership feeling [3], [4]. Also, the successful incorporation of an artificial limb is often shown by the fact that it evokes the same feelings as a real body part [4], for example participants might report feeling the touch [2], [20], [21] and pain [12], [22] in the rubber hand.

The discrepancy between the results of the studies on thermal pain during the RHI might be related to the unclear effect of pain on the sense of hand ownership. Although it is possible to induce the RHI with painful-tactile stimulation [22], it has also been shown that the RHI is less intense during pain stimulation than during illusion induction (during the brushing [12]). It is also possible, although speculative, that because applying noxious stimuli repeatedly to participant's hand and asking them each time to rate the pain intensity induces attentional task-set aimed at assessing the sensations from the real hand (e.g. [23]), and therefore reduces the effect of losing the ownership feeling towards the hand.

We decided to further investigate the effect of the RHI on the experience of thermal stimuli in the real hand with a different experimental protocol. There was no visible stimulation on the rubber hand that could strengthen the visual referral of pain to the rubber hand. We implemented a between-subjects design and applied the cold stimulus only once to avoid directing the attentional focus to the real hand due to the repeated sensory discrimination. This protocol also aimed to make participants completely naïve to the purpose of the study and to reduce the pain anticipation anxiety that might increase pain thresholds [24], [25]. We used an ice compress at around 0°C, which is usually experienced as unpleasant but it is not immediately painful [26]–[28]. The ice was applied to the area stimulated earlier with the brush, and it was not visible to the participants. Participants were asked to say “stop” when the stimulation became unpleasant. We hypothesised that altering the sense of ownership of the real hand would result in its reduced sensitivity to discomfort caused by cold stimulus, measured by participants' subjective ratings of unpleasantness and the time before they stop the ice application. We also used both subjective and behavioural measures of the RHI strength: ratings of rubber hand ownership, a questionnaire [2] and proprioceptive localization error, this being the degree to which a person misjudges the position of own unseen hand [8].

## Methods

### Participants

Following approval by an Ethics Committee in the Institute of Psychology, forty healthy volunteers participated in the study (28 females; mean age: 22.55, SD = 1.35). They all gave written consent. Participants were equally distributed to two groups: control and experimental. Participants were naïve to the purpose of the experiment and about some parts of the procedure: they were informed that they would be stroked on the hand and that the rest of instructions would be given later. They were informed they could resign from participation in the study at any moment.

### Materials

For the purpose of the experiment a wooden framework (120×60 cm) with two compartments divided by a vertical partition was built. The partition could be easily flipped horizontally and used as a framework cover. We used a natural-looking hand prosthesis to eliminate potential bias caused by artificial or non-corporal look of the rubber hand [29], [30], [31].

### Procedure

**Figure 1:**
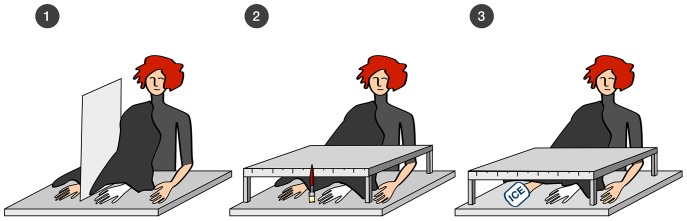
The experimental setup. (A) Hands were placed on two sides of the wooden wall during RHI induction. (B) Proprioceptive localization error was measured by moving a brush along the framework cover alongside a ruler that was not visible to the participant. (C) Ice compress was applied on the participant's right hand.

The experiment was carried out in a room of constant temperature (21°C). Participants were tested individually. The participant sat in front of the framework and placed his or her right arm inside the right compartment. The right rubber hand and the real left hand were placed in the left compartment in natural looking positions. The right arm and the forearm of the rubber hand were covered with cloth so they were not visible to the participant (Fig. 1A). The right hand was hidden for the whole duration of the experiment. The participants were asked to look at the rubber hand and to not move their real hands. The rubber hand and the participant's real hands were equidistant from the participant's body. The index fingers of the rubber hand and the right hand were 20 cm from each other and the distance between rubber hand and left hand was 40 cm.

#### RHI induction

The experimenter used two small brushes to stroke the fingers and dorsum of the participant's right hand and rubber hand. In the experimental group stroking was timely and spatially synchronized, whereas in the control group the stroking was spatially incongruent [14]. The stimulation lasted for three minutes. At the end of the session the participants were asked if they were experiencing an ownership feeling towards the rubber hand (1 – “I feel nothing” to 5 – “I feel as if the rubber hand were my hand”).

#### Proprioceptive localization error

After stroking, participants were asked to close their eyes and the whole box was covered so that all the hands were hidden. When participants opened their eyes they were asked about the position of their right hand. The experimenter moved a brush along the vertical part (the cover), starting 1 cm from the participants' left index finger, asking them to say “stop” when they thought the brush was located over their right middle finger (Fig. 1B). The distance between this point and the real position of the finger was measured (cm).

#### Cold sensitivity

Next, participants were warned that they might feel something unusual and asked to say “stop” when they started feeling uncomfortable. Then an ice compress taken directly from a portable freezer was applied to the dorsum of the participant's right hand and fingers and held in place by the experimenter. The time before the participant asked for the application to be stopped was measured (Fig. 1C). The maximum stimulation time was 120 s. Afterwards, the participant was asked to rate the unpleasantness of the experience on a 5-point scale (1–pleasant, 5–unpleasant). It is important to stress that all the hands were hidden during the cold stimulation and unpleasantness rating.

#### Questionnaire

At the end of the experiment participants were asked to complete a questionnaire measuring subjective RHI strength [2]. The questionnaire included 9 items with a scale ranging from “disagree strongly” (−3) to “agree strongly” (+3). The questionnaire was given to participants at the end of the experiment so as not to increase the amount of time between the induction of the illusion and cold discomfort measurement.

## Results

Analysis was conducted using standard statistical methods in SPSS software. The data of one participant was discarded from analyses of cold resistance time and unpleasantness intensity due to a lack of any declared discomfort or feeling of cold after 120 s of ice compress application.

### Rubber hand illusion

**Figure 2:**
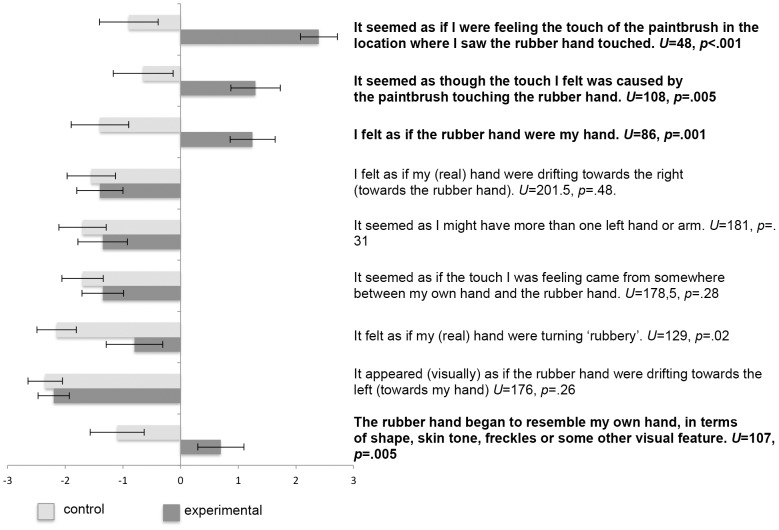
The mean level of agreement with the questionnaire statements in experimental and control groups.

A directional *t*-test for independent groups was conducted to compare the strength of the rubber hand illusion between the experimental and control group. We found no difference in feeling of rubber hand ownership between the experimental (M = 3.50, SD = .89) and the control group (M = 3.20, SD = .90) at the end of the stroking session, t(38) = 1.06, p = .14. However, there were differences between groups in the mean level of agreement to four questionnaire statements, analysed with a directional Mann-Whitney U-test (assuming the Bonferroni-corrected significance level  = .006). Participants in the experimental group (M = 1.25, SD = 1.74) agreed more strongly with statement 3 indicating feeling of hand ownership (“I feel as if the rubber hand were my hand”) than participants in the control group (M = −1.4, SD = 2.23), U = 86, p = .001. Participants also agreed more strongly with statement 1 (“It seemed as if I were feeling the touch of the paintbrush in the location where I saw the rubber hand touched”) in the experimental (M = 2.4, SD = 1.47) than in the control group (M = −.90, SD = 2.31), U = 48, p<.001. The difference in the mean level of agreement to statement 2 (“It seemed as though the touch I felt was caused by the paintbrush touching the rubber hand”) and statement 9 (“The rubber hand began to resemble my (own) real hand in terms of shape, skin tone, freckles or some on the visual feature”) were also statistically significant. The mean level of agreement to statement 2 in the experimental group (M = 1.30, SD = 1.94) was higher than in the control group (M = −.65, SD = 2.32), U = 108, p = .005, and similarly with statement 9 (experimental: M = .70, SD = 1.79; control: M = −1.1, SD = 2.12), U = 107, p = .005. All the results are presented on Figure 2.

**Figure 3:**
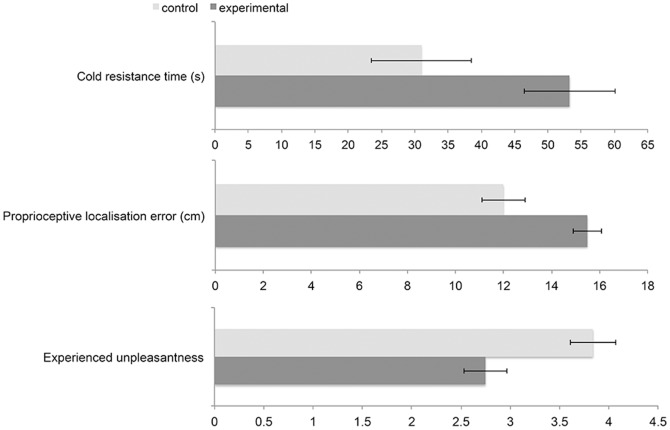
Differences in mean proprioceptive localisation error, cold resistance time and experienced unpleasantness between the experimental and control groups.

Proprioceptive localisation error occurred in both groups but was larger in the experimental group (M = 15.50 cm, SD = 2.70) than in the control group (M = 12.05 cm, SD = 3.94). This effect was statistically significant, t(38) = 3.22, p = .001 (Fig. 3). Additionally, we checked whether the localisation error was related to the feeling of rubber hand ownership (statement 3) across all participants. The Pearson correlation revealed the linear and positive relationship between the magnitude of the error and the strength of illusion, r = .44, p = .002.

### Cold-induced discomfort

Directional t-test comparisons for independent groups showed differences in resistance time to the cold compress: t(37) = 2.21, p = .02. Participants in the experimental group (M = 53.30 s, SD = 30.33) stopped the stimulation later than those in the control group (M = 31 s, SD = 32.60). Groups also differed in the level of unpleasantness they experienced, t(37) = −3.15, p = .001. Participants in the experimental group reported a lower level of unpleasantness (M = 2.75, SD = .97) than those in the control group (M = 3.74, SD = .99). These results are presented on Figure 3. Also, the two measures of cold sensitivity were related: we found a negative Pearson correlation between the level of unpleasantness and cold resistance time in the experimental group (r = −.77, p<.001) and control group (r = −.50, p = .01).

To investigate whether the cold-induced discomfort was related to an altered sense of limb ownership we conducted Pearson correlations with subjective and objective measures of the RHI separately for the experimental and control group. In the experimental group the magnitude of localisation error was negatively correlated with unpleasantness intensity (r = −.43, p = .03) and positively correlated with cold resistance time (r = .56, p = .005). In the control group we found correlation neither between the magnitude of localisation error and unpleasantness intensity (r = −.01, p = .48), nor between localisation error and cold resistance time (r = .14, p = .27).

The strength of agreement to questionnaire statement 3 correlated positively with cold resistance time among all participants (r = .30, p = .03) but not with experienced unpleasantness (r = −.25, p = .06). These correlations were statistically significant neither within the experimental group (statement 3 and cold resistance time: r = −.54, p = .41; statement 3 and unpleasantness: r = .16, p = .24), nor within the control group (statement 3 and resistance time: r = .30, p = .09; statement 3 and unpleasantness: r = −.14, p = .29).

## Discussion

In this experiment we showed that inducing the sense of rubber hand ownership reduces discomfort caused by cold stimulus applied to the real hand. This effect was shown by behavioural as well as subjective measures: participants in the experimental group requested the removal of the ice application later and rated the experience as less unpleasant compared to members of the control group. The intensity and the time of discomfort occurrence correlated with the proprioceptive localisation error in the experimental group. The time of discomfort correlated with the reported strength of the sense of rubber hand ownership across all participants.

The results are in line with the hypothesis linking alterations of body ownership to changes in the cortical systems maintaining homeostasis and interoception [4], [5]. The crucial role in this connection has been attributed to the insula – a structure involved in thermoregulation, thermoception and nociception [32]–[36] but also linked to the feeling of body ownership [4], [5], [35]–[37]. The integrative view was offered by Moseley [38] who introduced the concept of “body matrix” that is a representation of body and surrounding space linking regulatory functions such as temperature control and the prioritisation of tactile inputs to cognitive representation of body. Moseley [38] suggested that visuo-tactile conflict leads to recalibration of spatial body representation, in the case of RHI this means the inclusion of the rubber hand and exclusion of the real one. Due to connections between the posterior parietal cortex (involved in integration of spatial information) and the insular cortex this recalibration might result in impaired tactile, thermal and pain stimulation processing in the excluded hand. Taking proprioceptive localisation error for an index of recalibration of body representation we can say that in our experiment the stimuli was perceived as less unpleasant when the recalibration was stronger.

The full interpretation of the results requires consideration of the discrepancies between the results of this study and the previous studies on thermal pain perception during the RHI [12], [20], [21]. We think that there are two main factors, not necessarily mutually exclusive, that may play a role in these differences: the degree of real hand disownership induced in an experiment and the pain referral to the “owned” rubber hand.

Firstly, some data on the full-body illusion suggest that pain experience is reduced only when it is attributed to a body that is not felt as one's own. For example, an out-of-body illusion reduces skin conductance response to threats to one's real body [39] and the feeling of ownership of a virtual body correlates with increased pressure pain thresholds [40]. This interpretation would explain the weak analgesic effect in the RHI studies where the experimental and control conditions differed in the sense of the real hand disownership [12]. We cannot claim that the effect of cold sensitivity reduction in our experiment was caused by a sense of disownership of the real hand, as the subjective measures used in the experiment did not cover any direct questions about feelings towards the real hand. However, the strength of the rubber hand ownership (statement 3) did not correlate with the experienced unpleasantness while the magnitude of localisation error correlated with both, subjective and objective discomfort measures. We think that implying lack of ownership from first-person reports might be problematic [32] as in the context of RHI it might not be really experienced until a participant's attention is drawn to the limb. Taking that the feeling of ownership depends on body representation, we think that localisation error could be used as an indirect indicator of the real hand being excluded from this representation (although it is controversial, e.g. [18]).

Secondly, the previously reported lack of change in pain sensitivity in the real hand might have been due to the experimental protocol that made participants perceive the painful stimuli as being applied to the rubber hand (which was felt as their own body part during the experimental trials) [12], [20]. As a result, participants might have “felt” the same pain in both the control and experimental conditions, but in the latter the pain was “felt” in the rubber hand. It has been shown that the pain can be referred to the artificial hand [22] and that patients with a delusion of alien limb ownership report experiencing pain in the alien arm that they perceive as their own [41]. The referral of the pain to the rubber hand might have been strengthened by a visually salient heat probe attached to the rubber hand, raising expectations about the location of the upcoming painful event. Similarly, threatening the rubber hand elicits a threat-related neural response [4] and moving a hand towards the rubber hand raises expectations of touch [42]. This interpretation is in accordance with the result of this experiment in which there was no visible pain source on the rubber hand. It would also explain the increase of pain threshold in the study of Hegedüs and colleagues [21], as they placed the rubber hand on the heat probe built into the table, thereby reducing the salience of the painful stimuli and disturbing pain location attribution. However, this issue needs further study as it is not clear where the pain is felt in such a condition and why diminishing its location would influence the experience.

Thirdly, expectations of pain induced by a visible heat probe might enhance pain intensity in the experimental conditions in which participants see a heat probe on the “owned” rubber hand [12], [20]. Although it is still controversial (see e.g. [43]) some studies suggest that anxiety related to pain expectations and attention to pain might decrease pain thresholds [24], [25], [44], [45]. Also, Hofle and colleagues [46] showed that seeing the possible source of pain (a pin) touching a hand perceived as one's own induces higher intensity and unpleasantness ratings of electrical stimulation compared to just viewing the hand. However, at the same time one could expect that if the rubber hand became incorporated, then seeing it being stimulated with noxious stimuli would have an analgesic effect, as shown by Longo [47]. It is therefore also possible that these two effects diminish each other, but this clearly needs more exploration.

The presented study differs from previous studies in one other important aspect, namely that we measured cold-induced discomfort not thermal pain intensity and thresholds. Pain perception is thought to have at least two different components processed by separate cortical networks: the sensory-discriminative component that is related to perceived intensity and location and the affective-motivational component that reflects its unpleasantness [48]–[50]. It has been shown that it is possible to manipulate these aspects separately [51]. Moreover, unpleasantness is thought to be experienced at a lower threshold than pain and especially for cold stimuli the same thermal and pain intensity is rated as more unpleasant than for hot stimuli [52]. Although speculative, it is possible that unpleasantness is more susceptible than pain discrimination to psychological and contextual factors such as attention or perceived threat to health [53], [54]. For example, long-term meditators, compared to novices, are able to reduce unpleasantness but not perceived intensity of painful stimuli [53].

Although we believe that the results of the experiment show reduced discomfort to cold in the real hand after the RHI, the study has several limitations. Firstly, there was no difference between groups in feeling of rubber hand ownership just after the stroking, but the questionnaire revealed that this feeling was stronger in the experimental group in the retrospective judgement (at the end of the experiment). This surprising result might stem from the fact that the question asked at the end of the stroking session was too vague or confusing for participants. Another possibility is that the RHI induction did not elicit an immediate strong ownership experience and only after participants tried to locate their hand did they realise that they felt the rubber hand in front of them was their real hand. However, it is worth noting that subjective measures might not be a good way of capturing the sense of ownership since it is unclear whether participants report their feeling of ownership or their judgement of ownership [55]. Therefore, although the declared feeling of taking ownership of the rubber hand might not have been very strong, participants could have used some cues (i.e. difficulties with locating their hand) to judge their sense of ownership retrospectively. The second limitation of the study is the possibility that the effect of discomfort reduction was not limb-specific, as we did not measure it for other body parts. However, there seem to be no theoretical premises for expecting that it would generalise to the whole body, and the data suggest that such effects of RHI as pain reduction, histamine reaction or temperature drop are limited to the experimental hand [10], [12], [19]. It is still possible though, that the differences between the experimental and control group were due to some kind of attentional distraction related to the surprising vividness of the illusion in the experimental group [56], [57]. Another factor possibly responsible for the decrease of cold discomfort in the experimental group is the temperature drop in the experimental hand [10]. Unfortunately we cannot address this issue since we did not record limb temperature. However, this effect would support the hand disownership hypothesis revealing at least partially the mechanism of cold-related discomfort reduction.

To sum up, taking into consideration the growing evidence that altered body perception and body ownership can affect experienced pain level [12], [21], [40], [58], [59], we propose that our findings could be interpreted as indirect evidence of the possibility of pain and discomfort relief in the experimental hand under RHI. However, we think it is crucial to determine the conditions and mechanisms responsible for altering those experiences. This important area surely needs more exploration which, in our opinion, should include combined subjective and behavioural measures.
